# A foreign body that has been left for 20 years causes a pseudoaneurysm of the middle cerebral artery: a case report

**DOI:** 10.1186/s13256-023-04334-w

**Published:** 2024-02-01

**Authors:** Qiang Li, Zhengfang Jiang, Miao Yuan, Chenglang Xu, Lingyong Zeng

**Affiliations:** 1 Department of Neurosurgery, Affiliated Hospital of Southwest Medical University, Luzhou, 646000 Sichuan China; 2 Department of Neurosurgery, Sichuan Mianyang 404 Hospital, Mianyang, 621000 China

**Keywords:** Foreign object, Pseudoaneurysm, Middle cerebral artery, Subarachnoid hemorrage

## Abstract

**Background:**

We report a patient with extensive subarachnoid hemorrhage caused by the rupture of a middle cerebral artery pseudoaneurysm from a foreign body that had been left for two decades.

**Case presentation:**

A 74-year-old male patient from Han nationality was admitted to the emergency department of our hospital with impaired consciousness for 1 hour. Cranial computed tomography examination indicated a massive subarachnoid hemorrhage with intraventricular blood accumulation, and a high-density short strip dense shadow was seen in the M1 segment of the right middle cerebral artery, considering the possibility of a foreign body. Subsequently, a cerebral angiography was suggested; the foreign body was seen through the right middle cerebral artery, and the aneurysm was seen in the lower wall, so a pseudoaneurysm was considered. The emergent surgical intervention involved the clipping of the pseudoaneurysm and intracranial extraction of the foreign body. Unfortunately,the patient ultimately expired due to severe pulmonary infection.

**Conclusion:**

Intracranial pseudoaneurysm caused by foreign body has been rarely reported previously, and microsurgical treatment of an intracranial pseudoaneurysm caused by a foreign body is a good choice.

## Introduction

Intracranial pseudoaneurysms constitute a relatively uncommon pathological entity, accounting for approximately 1% of all intracranial aneurysms [1]. The etiology of pseudoaneurysms is multifactorial and encompasses a diverse range of causative factors, including: iatrogenic, traumatic, radiation-induced, vasculitic, infectious, and various other origins [1, 2]. Due to the elevated risk of rupture, expeditious implementation of either microsurgical intervention or endovascular treatment is frequently warranted.

## Case description

**Fig. 1:**
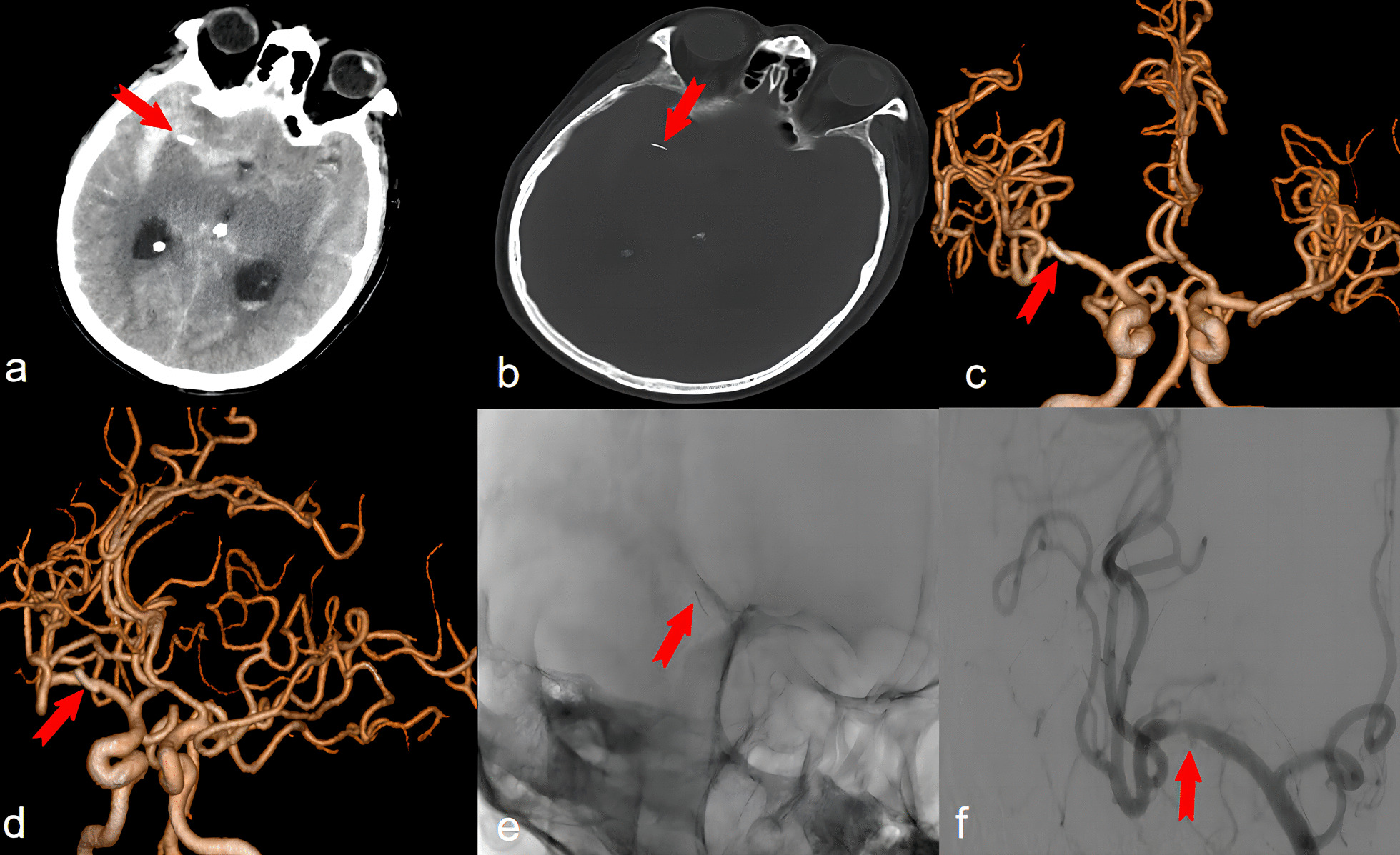
**a** Brain computed tomography scan showing diffuse subarachnoid hemorrhage; a short streak-like high-density shadow is seen next to the M1 segment of the right middle cerebral artery. **b** Bone computed tomography scan with evidence of a short strip-like dense shadow in the right cerebral hemisphere. **c**, **d** Computed tomography angiography (CTA) of the head and neck shows calcified plaques with severe luminal stenosis and foreign body presence at the M1 bifurcation of the right segment of the middle. **e**, **f** Cerebral angiogram showing a foreign body penetrating the middle cerebral artery with a saccular aneurysm arising from the inferior wall, which was considered a intracranial pseudoaneurysm. The red arrow in **a**–**f** indicates a foreign body

**Fig. 2:**
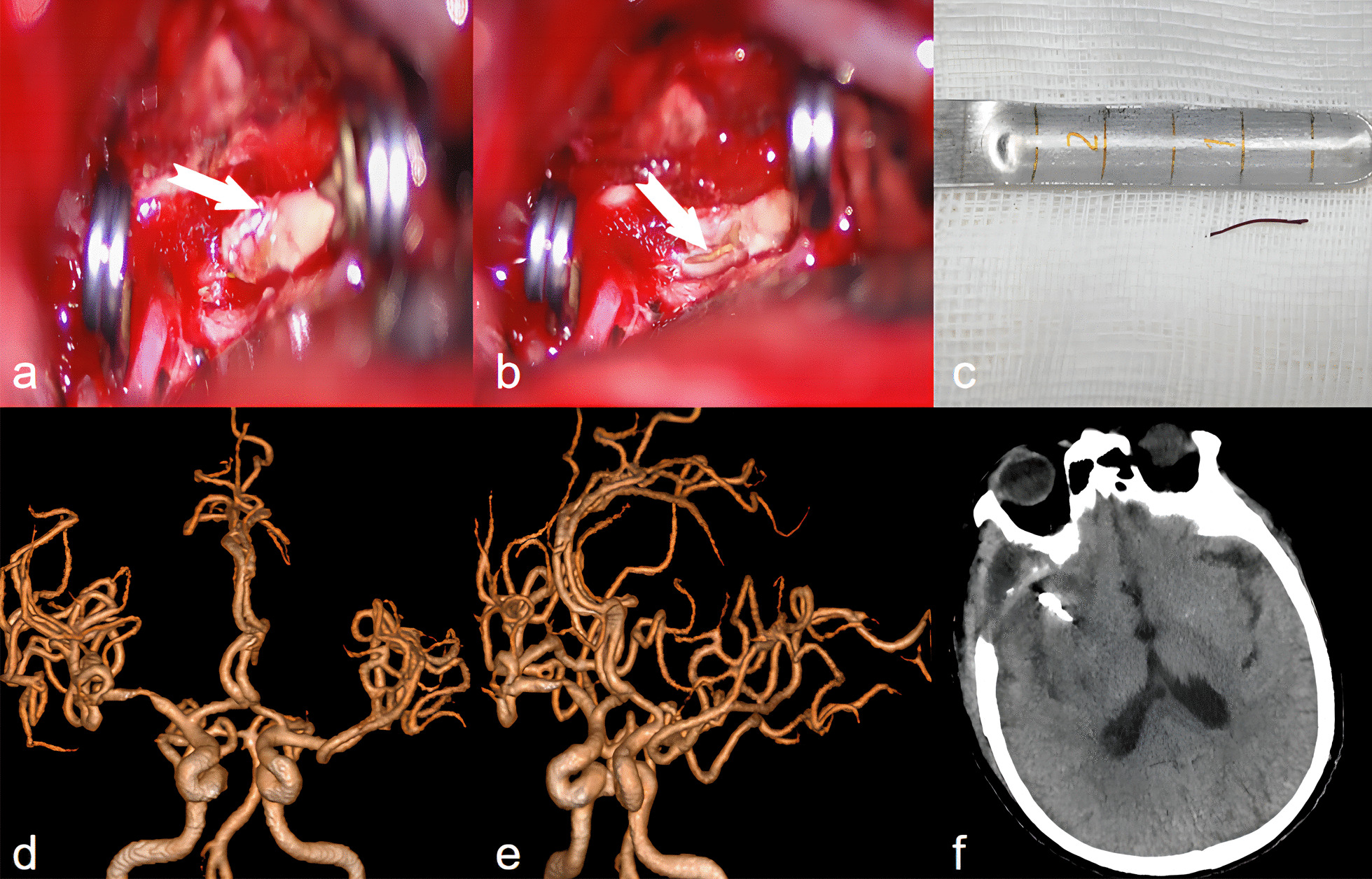
**a** A temporary block of the proximal and distal M1 segment of the middle cerebral artery, and a clip of the aneurysm neck with a 6.5 mm curved aneurysm clip. **b** Microscopic inspection revealed a golden-colored metallic foreign body approximately 1 cm in length, situated within the M1 segment of the right middle cerebral artery. **c** The foreign body is about 1 cm and covered with rust. **d**, **e** Postoperative computed tomography angiography (CTA) showed that the foreign body was removed. **f** Intracranial CT scan 10 days after surgery showed significant absorption of subarachnoid hemorrhage. The white arrows in **a** and **b** indicate the location of the foreign object under the microscope

We present a unique and intricate case involving a 74-year-old male patient from Han nationality who was urgently admitted to our emergency department with a 1-hour history of altered consciousness. The patient had no prior high-risk vascular factors, significant head trauma, or surgical history. Upon initial assessment, the Glasgow coma scale (GCS) score was 4, with positive signs of meningeal irritation. Emergency cranial CT imaging revealed a substantial subarachnoid hemorrhage accompanied by a intraventricular hemorrhage. Additionally, a high-density linear metallic opacity was observed within the M1 segment of the right middle cerebral artery, raising suspicion of a foreign body (Fig. 1a and b). Further inquiry with the patient’s family revealed a history of head acupuncture therapy performed two decades earlier, associated with transient episodes of headache. A head and neck computed tomography angiography (CTA) revealed calcified plaques and significant narrowing of the vessel lumen and the presence of a foreign body at the M1 bifurcation of the right segment of the middle cerebral artery (Fig. 1c and d). A subsequent meticulous cerebral angiography confirmed the penetration of a foreign body within the right middle cerebral artery, with an associated pseudoaneurysm formation on the undersurface of the arterial wall (Fig. 1e and f). Emergent microsurgical intervention was deemed necessary, during which a metallic foreign body, approximately 1 cm in length, was meticulously extracted from the M1 segment of the right middle cerebral artery. The foreign body, observed to be metallic and gold colored, had one end embedded approximately 3 mm into the arterial wall (with a slightly pointed tip) and approximately 3 mm exposed within the surrounding inflammatory tissue (Fig. 2b and c). Adjacent to the point of vascular entry, a pseudoaneurysm was evident. The therapeutic strategy involves using a microsurgical clip to address a pseudoaneurysm in the M1 segment of the right middle cerebral artery. encapsulation of the pseudoaneurysm will be performed, and any foreign bodies will be removed during the procedure. (Fig. 2a). Subsequent postoperative computed tomography angiography (CTA) showed that the foreign body was removed (Fig. 2d and e). Ten days after surgery, cranial CT scans revealed significant absorption of the subarachnoid hemorrhage (Fig. 2f), and ventilator support was discontinued. Regrettably, the patient developed a severe pulmonary infection, possibly precipitated by the sudden loss of consciousness, which ultimately resulted in his demise 3 weeks postoperatively.

## Discussion

This case serves as an important contribution to medical literature as the first documented instance of an intracranial pseudoaneurysm induced by an intracranial foreign body. Given the patient’s comatose state upon admission and the inability to obtain a comprehensive medical history, it underscores the critical role that clinical history and advanced imaging play in the accurate diagnosis and management of such intricate cases.

Intracranial pseudoaneurysms remain a rare and highly lethal condition. While previously reported cases often cite etiological factors, such as iatrogenic factors, traumatic factors, or infectious origins [1], instances where foreign bodies precipitate intracranial pseudoaneurysms are exceptionally uncommon. The foreign body in question may encompass emboli, remnants of medical devices, or other materials inadvertently introduced into cerebral arteries during medical procedures, including foreign metallic objects. The clinical presentation of intracranial pseudoaneurysms induced by foreign bodies can vary significantly based on the pseudoaneurysm’s location and size and the root cause of foreign body introduction. Common symptoms may encompass severe headaches, neurological deficits, epileptic seizures, and, in severe cases, intracranial hemorrhage. As such, timely diagnosis and intervention are paramount in preventing potential catastrophic outcomes.

The precise diagnosis of intracranial pseudoaneurysms induced by foreign bodies typically involves a combination of medical imaging techniques. Computed tomography angiography (CTA) and magnetic resonance angiography (MRA) are invaluable tools for visualizing vascular abnormalities and identifying the presence of pseudoaneurysms. A digital subtraction angiography (DSA) provides real-time vascular images and is considered the gold standard for diagnosis [3], aiding in pinpointing the exact location and characteristics of the pseudoaneurysm [4]. The management of intracranial pseudoaneurysms induced by foreign bodies often necessitates a multidisciplinary approach, involving neurosurgeons, interventional radiologists, and other specialized medical professionals. Treatment strategies depend on factors such as the pseudoaneurysm’s size and location, the patient’s overall health, and the underlying cause of foreign body introduction [5]. Endovascular coiling is suitable for managing smaller pseudoaneurysms located in accessible regions. This method utilizes endovascular techniques, specifically coil embolization, wherein coils are inserted into the pseudoaneurysm to impede blood flow and mitigate the risk of rupture [6]. On the other hand, surgical clipping is reserved for larger or intricate pseudoaneurysms that necessitate open surgical intervention. Neurosurgeons may perform surgical clipping to isolate and protect the affected vessel, preventing further bleeding [7]. In cases where the pseudoaneurysm is directly associated with a foreign body, such as remnants of emboli or fragments from medical equipment, the extraction of the foreign object is a crucial component of the treatment process. Post-treatment surveillance is imperative, encompassing the continuous monitoring of interventions and close observation for potential complications, such as rebleeding, neurological deficits, pulmonary infections, etc. Follow-up imaging examinations play a crucial role in confirming the successful treatment of pseudoaneurysms.

## Conclusion

Intracranial pseudoaneurysms induced by foreign bodies represent a challenging and rare pathological entity, necessitating prompt diagnosis and intervention. The rarity of such occurrences underscores the critical importance of maintaining a high index of suspicion, particularly in cases involving recent medical trauma or craniocerebral injuries. Simultaneously, timely therapeutic interventions can significantly enhance patient outcomes and mitigate the risks of life-threatening complications.

## Data Availability

Not applicable.
